# Effects of bacterial lipopolysaccharides on platelet function: inhibition of weak platelet activation

**DOI:** 10.1038/s41598-020-69173-x

**Published:** 2020-07-23

**Authors:** Alexey A. Martyanov, Aleksandr S. Maiorov, Aleksandra A. Filkova, Alexander A. Ryabykh, Galina S. Svidelskaya, Elena O. Artemenko, Stepan P. Gambaryan, Mikhail A. Panteleev, Anastasia N. Sveshnikova

**Affiliations:** 1 Center for Theoretical Problems of Physico-Chemical Pharmacology, Russian Academy of Sciences, 30 Srednyaya Kalitnikovskaya str., Moscow, 109029 Russia; 2 National Medical Research Center of Pediatric Hematology, Oncology and Immunology Named After Dmitry Rogachev, 1 Samory Mashela St., Moscow, 117198 Russia; 3 Institute for Biochemical Physics (IBCP), Russian Academy of Sciences (RAS), Kosyigina 4, Moscow, 119334 Russia; 4 Faculty of Physics, Lomonosov Moscow State University, 1/2 Leninskie Gory, Moscow, 119991 Russia; 5 Sechenov Institute of Evolutionary Physiology and Biochemistry, Russian Academy of Sciences, St. Petersburg, 194223 Russia; 6 Department of Normal Physiology, Sechenov First Moscow State Medical University, 8/2 Trubetskaya St., Moscow, 119991 Russia

**Keywords:** Platelets, Inflammation

## Abstract

Platelets are anucleate blood cells with reported roles in hemostasis and immune responses, which possess a functional receptor for bacterial lipopolysaccharides (LPSs), the well-known inducers of inflammation. However, LPSs effects on platelets are contradictory. Here we aim to investigate mechanisms of platelet functioning in the presence of LPS and to find the cause of the discrepancy in the previously published data. Cell activity was analyzed by flow cytometry, western blotting, and aggregometry. Thrombus growth was assessed by fluorescent microscopy. LPS' activity was checked by their capability to induce PMN activation. However, LPSs did not substantially affect either thrombus growth in flow chambers, irreversible platelet aggregation, or platelet responses to strong activation. Platelet aggregation in response to 1 μM of ADP was significantly inhibited by LPSs. Flow cytometry analysis revealed that platelet activation responses to weak stimulation were also diminished by LPSs, while VASP phosphorylation was weakly increased. Additionally, LPSs were capable of inhibition of ADP-induced P2-receptor desensitization. Incubation of platelets with a pan-PDE inhibitor IBMX significantly enhanced the LPSs-induced platelet inhibition, implying cAMP/cGMP dependent mechanism. The discrepancy in the previously published data could be explained by LPS-induced weak inhibition of platelet activation and the prevention of platelet desensitization.

## Introduction

Lipopolysaccharides (LPSs) are the components of the outer membrane of the gram-negative bacteria^1^, which are recognized by the toll-like receptor 4 (TLR4)^2^. LPS are among the most potent mediators of bacteria-induced sepsis and associated disseminated intravascular coagulation (DIC)^3^. TLR4 was detected on the surface of myeloid cell line: granulocytes, monocytes, dendric cells^4^. TLR4 is also expressed on the surface of platelets^5,6^—anucleate blood cells, responsible for hemostasis^7^. In order to recognize LPS, TLR4 requires such cofactor proteins as CD14, myeloid-differentiation factor 2 (MD2), and LPS binding protein (LBP)^8^. Upon ligation and dimerization, TLR4 induces the formation of a "myddosome" signaling complex, which initiates nuclear factor kappa-B (NF-κB) intracellular signaling cascade^2,8,9^. In anucleate platelets, the most important part of this cascade is the inhibitor of NF-κB (IκB), which has been reported to mediate granule secretion and other signalling events^8^.

LPS mediated TLR4 activation is claimed to enhance platelet stimulation by ADP and CRP^6,10,11^. LPS also were shown to induce increased oxygen consumption by platelet mitochondria^5,12^. LPS driven platelet activation is a potential mediator of neutrophil extracellular traps (NETosis) in vivo^8^. Finally, it was reported that LPS are capable of inducing synthesis of IL1β in platelets^13^. Platelet reactivity to LPS could be caused by activation of IKκ-β involved in phosphorylation of synaptosomal-associated protein 23 (SNAP23), which controls platelet alpha-granule release^8^. However, this was in disagreement with LPS capability to enhance aggregation of washed platelets, stimulated by thrombin, but without any effect on aggregation in platelet-rich plasma (PRP)^6^. Furthermore, there is a considerable amount of data that LPS effects on platelets are elusive^5,14^.

Besides the potentiation of cell responses to activation, LPS via TLR4 can act the other way around and inhibit cell responses. LPSs induce an increase in cAMP levels in various cell types^15,16^, while cAMP inhibits platelet activation^17^. One potential mechanism of this is TLR4 induced adenyl cyclase 3 (AC3) activation, demonstrated in bladder epithelial cells^18,19^. It is noteworthy that AC3 is also present in human platelets^20^. On the other hand, it has been shown that LPSs induces cGMP production in platelets^11^ in a TLR4/MyD88 dependent manner. However, authors claim that cGMP has a stimulatory role which is contradictory, to generally accepted theory that cGMP/PKG pathway plays inhibitory role in platelets^21^.

Here we aim to analyse the LPS effects on platelet responses to physiological activation. We show, that LPS could induce polymorphonuclear (PMN) cells activation and platelet-PMN heteroaggregate formation. LPS effects on platelet functioning ex vivo were limited to a slight increase in platelet thrombus height. Analysis of the platelet intracellular signalling in vitro revealed that LPSs were able to partially compensate ADP induced P2Y1 receptor desensitisation upon exposure to low doses of ADP. A more thorough analysis of the LPS effects on platelet signalling revealed that LPSs are capable to inhibit weak platelet activation via cAMP/cGMP signalling pathways. This phenomenon might be the key to understanding the inconsistency in the data on LPS mediated platelet activity.

## Results

### LPSs induced PMN activation but did not affect platelet aggregation in citrated plasma

**Figure 1:**
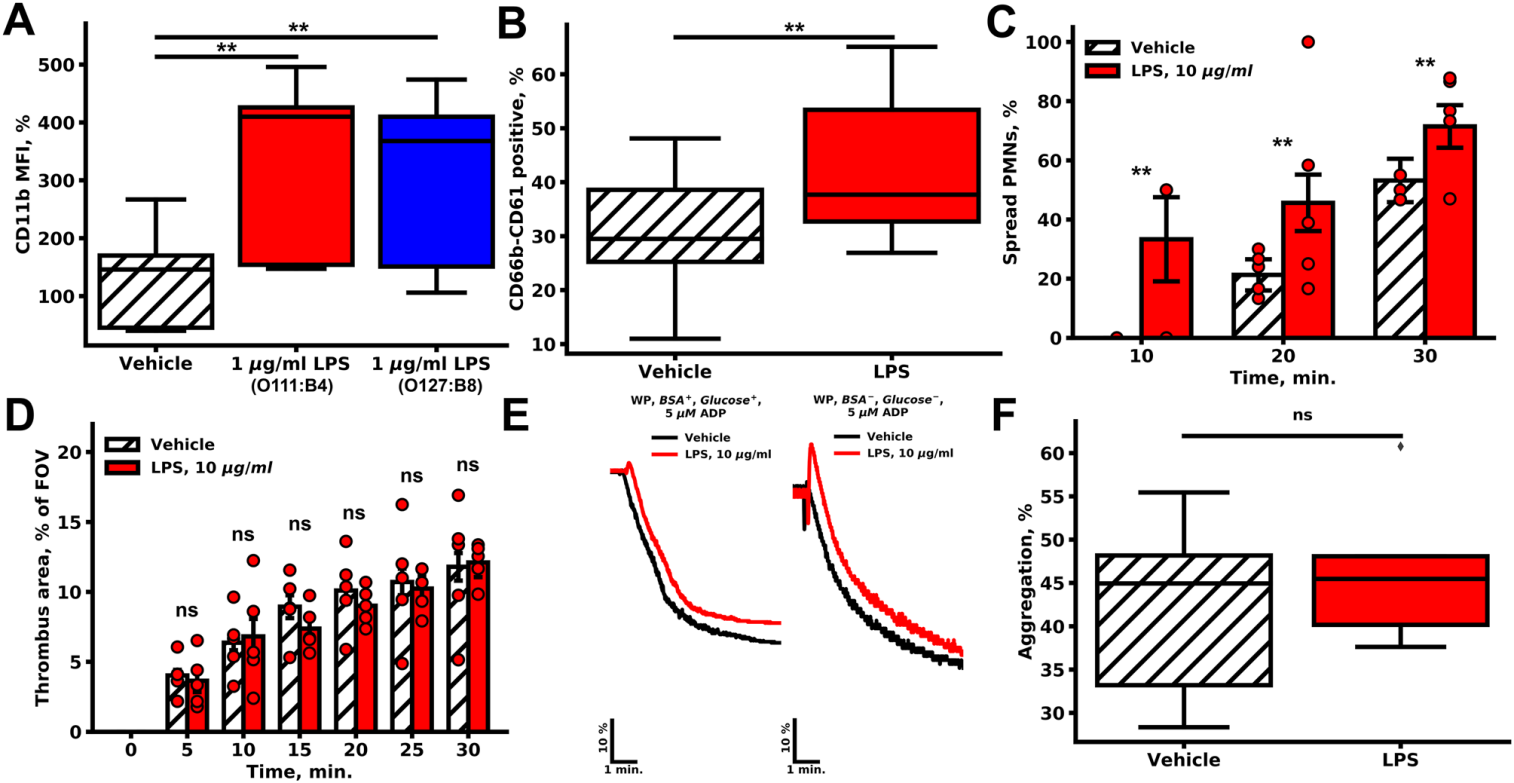
Effects of LPSs on PMN and platelet activity. (**A**, **B**) Flow cytometry of platelet and PMN suspension. (**A**) Pre-incubation with LPSs of different origins (red—1 µg/ml E. coli O111:B4, blue—1 µg/ml *E. coli* O127:B8) resulted in the increased CD11b fluorescence in comparison to the vehicle (n = 10). (**B**) LPSs increased the amount of CD66b-CD61 positive events (n = 10). (**C**, **D**) For microscopy experiments, whole blood was perfused through parallel-plate flow chamber with fibrillar collagen coating (spread PMN and thrombus area quantification is described in methods); (**C**) LPSs significantly increased the amount of highly spread PMNs (n = 5); (**D**) pre-incubation with LPSs non-significantly decreased thrombus area at wall shear rate 200 s^−1^ (n = 5). For (**C**, **D**) bars represent mean, whiskers represent SEM, significance was calculated by Mann–Whitney test, n = 5, **p < 0.01; ***p < 0.001. (**E**–**F**) Light transmission aggregometry; (**E**) characteristic aggregation curves of washed platelets (n = 5); (**F**) Maximum platelet aggregation in citrated PRP upon stimulation with 5 μM of ADP. Statistical significance was calculated by the Wilcoxon test, n = 10.

LPSs were reported to lose activity during storage. To test the activity of LPSs, we performed flow cytometry assessment on washed PMNs ability to induce CD11b activation upon pre-incubation with LPSs of different origins (Figs. 1A, S1A,B), which in contrast to reported data was independent on the presence of albumin. Additionally, LPSs significantly increased the amount of platelet-PMN heteroaggregates (CD61-CD66b double-positive events) in the washed cell suspension (Figs. 1B, S1C,D). To determine whether activated by LPSs PMNs have an impact on thrombus formation, we performed parallel-plate flow chamber thrombus growth experiments with fibrillar collagen type I^22,23^ (Figs. 1C,D, S2). Thrombus height and area, as well as parameters of the PMN activity, were estimated. For PMNs, pre-incubation with LPS resulted in an increase in PMN activation both in velocity (Fig. S2A) and the number of spread cells (Figs. 1C, S2B). These observations are in agreement with the hypothesis that LPS induce PMN activation in whole blood.

The LPS-activated PMNs could have an impact on platelet aggregation and thrombus formation in whole blood. Thrombus growth on fibrillar collagen was not significantly altered (Figs. 1D, S2D,E). LPSs did not significantly influence light transmission platelet aggregometry in various conditions such as PRP in citrated plasma, or washed platelets stimulated with ADP or TRAP, in the presence or absence of albumin, sCD14 or LBP (data not shown) in the solution, incubation with LPSs for 30–120 min at 25 °C or 37 °C. (Figs. 1E,F, S3). Thus, we concluded that LPS did not alter thrombus growth or platelet aggregation.

### Platelet functional responses to conventional activation were not affected by LPSs in vitro

**Figure 2:**
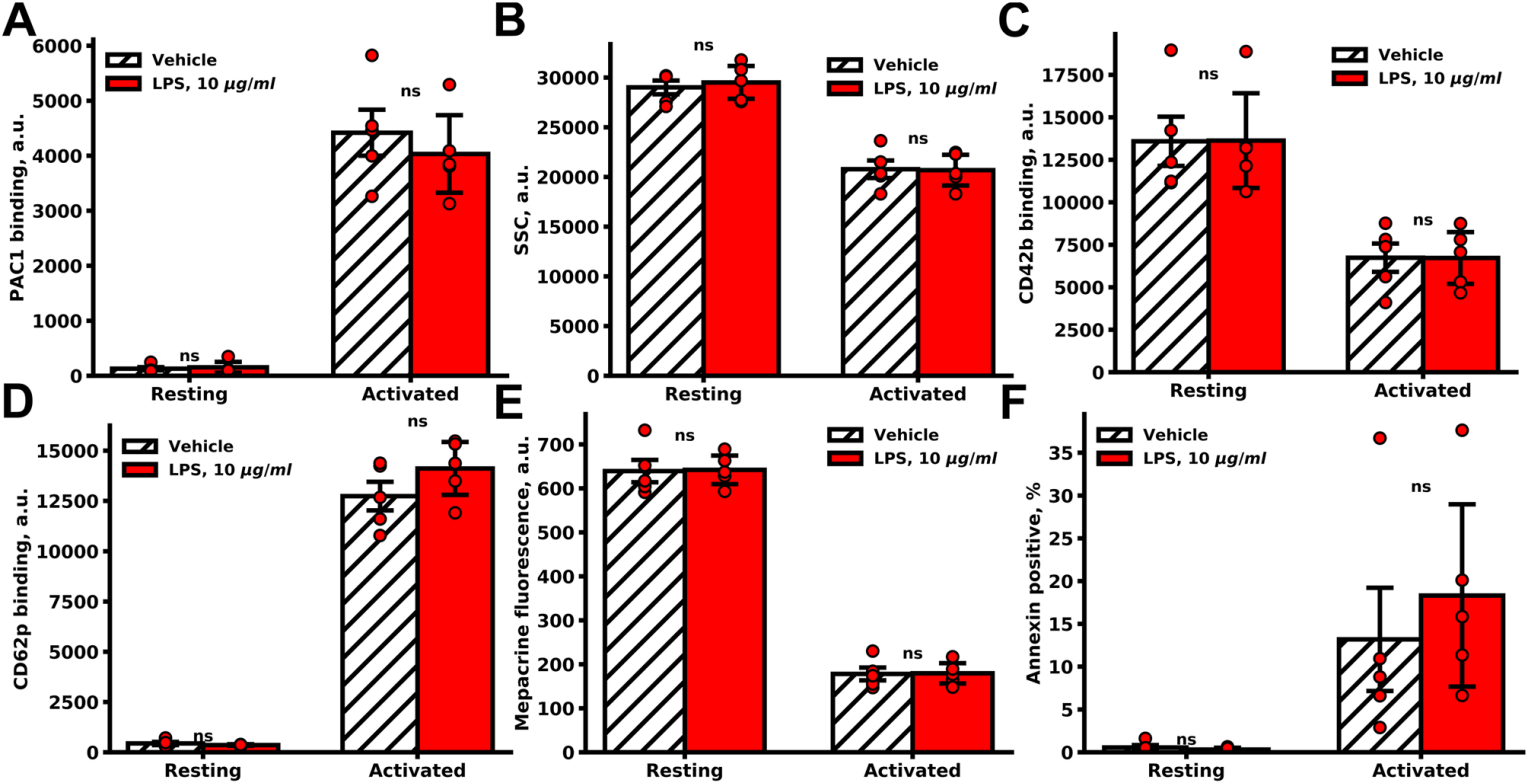
LPS did not significantly influence platelet functional responses. End-point flow cytometry analysis of washed platelets incubated with LPS. Platelets were pre-incubated with LPS (10 µg/ml LPS O111:B4, 30 min) and analyzed for PAC1 binding (**A**), shape change (SSC, **B**), CD42 shedding (**C**), CD62p exposure (**D**), dense granule secretion (mepacrine fluorescence **E**), Marker of platelet apoptosis (Annexin-V positive platelets **F**) upon strong activation by 20 µg/ml of CRP and 25 µM TRAP-6 for 10 min (n = 5). (**D**–**F**) Bars represent means. Whiskers represent SD. Significance was calculated by Mann–Whitney test, *p < 0.05; **p < 0.01; ***p < 0.001.

Application of end-point flow cytometry^24^ methods for in vitro analysis of platelet functional responses showed that LPS had minimal effects on platelet activation. Incubation with LPSs for 30–120 min did not alter either platelet integrin activation, shape change, GP1b shedding, α, and δ-granule release, and PS exposure upon stimulation with PAR1 activating peptide (TRAP) and CRP (Fig. 2). Keeping in mind that TLR4 stimulates the NFκB pathway, we tested IKκB phosphorylation in platelets and PMNs (Fig. S4). While incubation of washed PMNs with 10 µg/ml of LPS for 10 min resulted in significant IKκ-β phosphorylation, platelet IKκ-β phosphorylation under the same conditions was not affected (Fig. S4).

### LPS was involved in compensation of ADP-receptor desensitization

Platelets have two ADP receptors: P2Y_1_ (G_q_-associated, induces cytosolic calcium response) and P2Y_12_ (G_i_-associated, reduces cytosolic platelet cAMP)^7,25^. As well as other GPCR receptors, platelet P2Y_1_ internalizes upon activation in the β-arrestin and protein-kinase C (PKC) or G-protein coupled receptor kinase (GRK) dependent manner^26–29^. Also, the receptor reinternalization is mediated by serine-phosphatases, such as protein phosphatase 2A (PP2A)^29–31^, which was found in platelets^20^. Hereafter, we analyzed the LPS effect on platelet calcium signaling. Whole blood was pre-incubated with (1) LPSs, (2) LPSs and sCD14, (3) 100 nM ADP, and (4)100 nM ADP and LPSs and activated by low doses of ADP (Figs. S5, S6), or by CRP or PAR1 activating peptide (Fig. S7). Changes of cytosolic calcium concentration and fibrinogen binding were analyzed by flow cytometry. Pre-incubation (30 min) with 100 nM ADP resulted in a decrease in basal calcium concentration, which was restored to normal levels by LPSs (Fig. S5B). Neither sole LPS nor LPS with sCD14 influenced platelet calcium responses to ADP or other agonists (Figs. S5, S6). On the other hand, LPSs were capable of restoring the ADP-induced calcium mobilization in desensitized to ADP platelets upon stimulation by 250 or 500 nM of ADP (Figs. S5C and S6E, correspondingly). Fibrinogen binding has been restored as well (Fig. S6C,D). For higher ADP concentrations, the effects of LPS became non-significant for calcium (Figs. S5E,F, S6A). Nevertheless, fibrinogen response still could be restored by LPS (Fig. S6E,F). Also, CRP-induced fibrinogen binding was diminished upon LPS introduction in all combinations (Fig. S7E).

**Figure 3:**
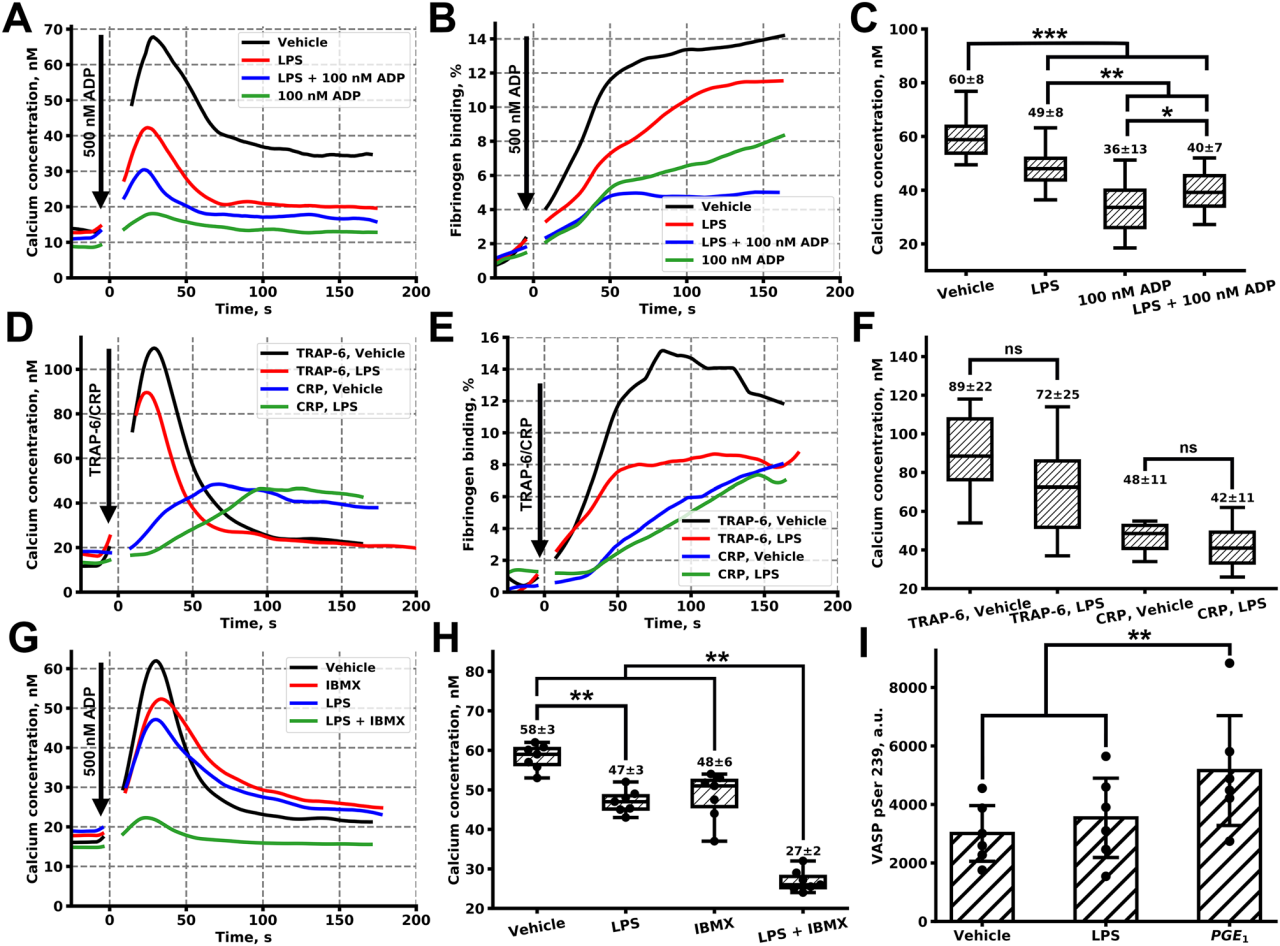
Analysis of impact of LPSs on platelet signalling responses in diluted blood plasma. (**A**, **B**) Typical cytosolic calcium concentration (**A**) and fibrinogen binding (**B**) curves upon activation by 500 nM ADP after pre-incubation with 100 µg/ml LPS, 100 µg/ml LPS and 100 nM ADP, 100 nM ADP or vehicle. (**C**) Pretreatment with LPS, LPS and ADP or sole ADP resulted in a significant decrease in a calcium response to 500 nM ADP (n = 15 donors, significance was calculated by Mann–Whitney test). (**D**, **E**) Typical cytosolic calcium concentration (**A**) and fibrinogen binding (**B**) curves upon activation by 1 µM TRAP-6 or 1 µg/ml CRP after pre-incubation with 100 µg/ml LPS or vehicle. (**F**) LPS pretreatment resulted in a non-significant decrease in calcium response to TRAP-6 and moderately altered calcium response to CRP (n = 15 donors, significance was calculated by Mann–Whitney test, *p < 0.05; **p < 0.01; ***p < 0.001). (**G**) Typical cytosolic calcium concentration curve upon activation by 500 nM ADP after pre-incubation with 10 µM IBMX, 100 µg/ml LPS or 10 µM IBMX and 100 µg/ml LPS simultaneously. (**H**) Platelet pretreatment with sole LPS or IBMX resulted in a weakly decreased calcium response to 500 nM ADP, LPS and IBMX significantly decreased calcium response to ADP (n = 7, significance was calculated by Mann–Whitney test, *p < 0.05; **p < 0.01; ***p < 0.001). (**I**) VASP pSer 239 phosphorylation assay by means of flow cytometry revealed non-significant increase in pVASP phosphorylation after 100 µg/ml LPS pre-treatment and a significant increase upon stimulation by 5 µM PGE_1_ (n = 6, significance was calculated by Mann–Whitney test, *p < 0.05; **p < 0.01; ***p < 0.001, whiskers represent SD).

In order to reduce the influence of other blood cells on LPSs concentration, we had performed additional experiments when 50-times diluted hirudinated PRP was pre-incubated with LPS, 100 nM ADP or LPSs and 100 nM ADP. In these experiments, calcium concentration in resting platelets was unaltered (Fig. S8A); however, calcium mobilization and fibrinogen binding were significantly diminished by LPS upon activation with 500 nM ADP (Figs. 3A–C, S8, p < 0.001). Pre-treatment with the desensitizing concentration of ADP (100 nM) resulted in a more significant decrease of both calcium and fibrinogen response (Figs. 3A–C, S8B, p < 0.001), however, the decline in platelet responsiveness to low concentrations of ADP was less pronounced in the presence of the combination of ADP and LPS (Figs. 3A–C, S8B, p < 0.05). These effects preserved, yet became less significant, upon activation by 1 µM ADP as well (Fig. S8D–F, p < 0.01). Pre-treatment of platelets with LPSs also resulted in the non-significant, yet a detectable decrease of platelet responsiveness to 1 µM of TRAP-6, and a moderate alteration in platelet responsiveness to 1 µM of CRP (Figs. 3D–F, S8C).

### *LPS diminish weak platelet activation *via* cAMP/cGMP signaling pathways*

In order to investigate the mechanism of LPS-mediated inhibition of platelet responsiveness to weak stimulation, we have pre-treated platelets with 10 µM of pan-PDE inhibitor IBMX for 30 min. IMBX pre-treatment results in the reduction of the PDE mediated cAMP/cGMP concentration increase and, thus, potentiate cAMP/cGMP cell responses^32^. At this concentration, IBMX did not completely inhibit platelet activation by 500 nM of ADP (Fig. 3G,H); however, the combination of 10 µM IBMX with 100 µg/ml LPSs resulted in significant impairment of platelet calcium response to 500 nM of ADP (Fig. 3G,H, p < 0.01). This result implies a synergistic effect of LPS and IBMX, based on an LPS-induced weak cAMP/cGMP rise, enhanced by IBMX mediated PDE inhibition. Next, we analyzed the activity of cyclic nucleotide-dependent kinases using the flow cytometry analysis of vasodilator-stimulated phosphoprotein (VASP) phosphorylation (on Ser 239). Pre-treatment of hirudinated PRP with 100 µg/ml of LPS resulted in a non-significant, yet detectable increase in VASP phosphorylation (Fig. 3I), which also could be induced by an LPS-induced weak cAMP/cGMP rise.

**Figure 4:**
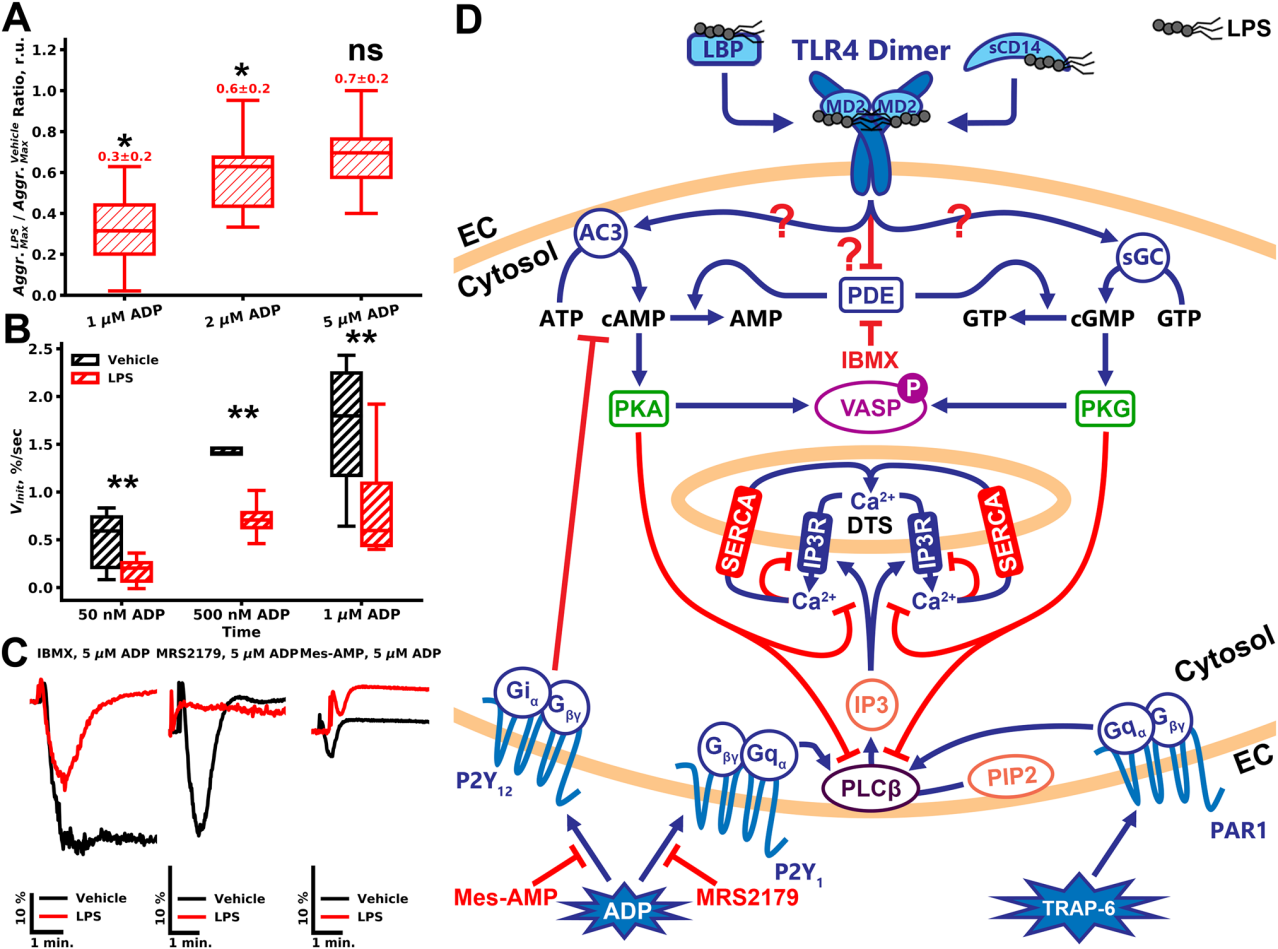
Impact of LPSs on weak platelet activation. (**A**) Aggregation of hirudinated PRP was significantly impaired upon LPS pretreatment in comparison to vehicle-treated PRP upon activation by 1 or 2 µM of ADP, while stronger stimulation was not significantly decreased (n = 15, significance was calculated by Mann–Whitney test, *p < 0.05; **p < 0.01; ***p < 0.001). (**B**) LaSca analysis also revealed LPS-induced impairment of the platelet aggregate formation after stimulation by 50 nM, 500 nM, and 1 µM of ADP in comparison to vehicle-treated PRP. (**C**) LPS completely inhibited platelet aggregation in the presence of P2Y_1_ receptor inhibitor MRS 2179 and moderately altered platelet aggregation in the presence of a P2Y_12_ inhibitor. IBMX itself did not alter aggregation in the vehicle (PBS) treated platelets. However, in the presence of IBMX, LPS more effectively inhibited platelet aggregation to 5 µM of ADP. (**D**) The proposed scheme of LPS influence on platelet activation. LPSs in the presence of plasma proteins (sCD14 and LBP) via platelet TLR4 receptor induce an increase in platelet cytosolic cAMP/cGMP. This increase can be mediated by activation of AC3, sGC, or by the inactivation of the PDEs. cAMP/cGMP production results in the PKA or PKG activation, which, in turn, are capable of inhibiting PLCβ, which produces IP_3_ from PIP_2_ upon activation by Gq. PKA/PKG also inhibits IP3R, which releases calcium ions from platelet calcium stores (*DTS* dense tubular system). Calcium mediates most of the platelet functional responses, such as integrin activation, granule release, shape change, and procoagulant activity.

To test the capability of LPSs to alter platelet functional responses upon weak stimulation, we performed additional aggregation assays in hirudinated PRP (Figs. 4A, S9), where platelet activation responses are weaker than in citrated PRP^33^. At all concentrations of ADP tested (1 µM, 2 µM, 5 µM, Fig. S9A–C, correspondingly) LPS inhibited PRP aggregation. The inhibition of aggregation by LPS was observed only when the aggregation was reversible (PRP in the presence of calcium ions). However, this effect was significant only for low (250 nM and 500 nM of ADP) concentrations of ADP (Fig. 4A, p < 0.05). Platelet di-aggregate formation in response to sub-threshold doses of ADP (50 nM, 500 nM) also was impaired by LPSs (Figs. 4B, S9D, p < 0.01). Inhibitors of platelet ADP receptors P2Y_1_ (MRS2179) and P2Y_12_ (Mes-AMP) significantly decreased platelet aggregation to 5 µM of ADP (Fig. 4C). Pre-incubation with LPS decreased platelet response to ADP in MRS2179 treated PRP (Fig. 4C). On the other hand, LPSs' effect on Mes-AMP treated PRP was moderate, yet detectable (Fig. 4C). Only combined with LPS, IBMX significantly decreased platelet aggregation in response to ADP (Fig. 4C), which also implies cAMP/cGMP-dependent mechanisms of platelet LPS-induced activation.

## Discussion

A thorough analysis of LPS effects on platelets and determination of the nature of discrepancies in available experimental data were the prime goals of this study. Here we demonstrate that LPSs induce PMN activation (Figs. 1, S1), which was in a perfect agreement with the literature data^34^. Furthermore, LPS increased IKκB phosphorylation in PMN (Fig. S4A), and the amount of platelet-PMN heteroaggregates in the washed cell suspension (Fig. 1B). However, LPS did not induce IKκ-β phosphorylation in platelets (Fig. S4B) and did not significantly affect neither thrombus growth (Figs. 1D, S2D,E) nor conventional platelet aggregation (Figs. 1E, S3). Analysis of platelet functional responses revealed no significant effect of LPS on platelet activation by strong (Fig. 2A–C) or intermediate stimulation (Fig. S7). This corresponded with the results from^5,14,35^, while being in disagreement with^6,10,12^. Using continuous flow cytometry of platelet activity upon weak stimulation, we determined that incubation with LPS was capable of inhibition of platelet responses to weak activation potentially via cyclic-nucleotide dependent signaling pathways (Figs. 3, 4). This phenomenon might be the key to understanding the inconsistency in the data on LPS mediated platelet activity.

Although LPS-induced activation of PMNs and macrophages has been demonstrated both in vitro^36^ and in vivo^37^, we have verified this ability of LPS in order to ensure that they are active. In our experiments, the height of the growing thrombi was increased in the presence of LPS (Fig. S2E). This effect, however, could be explained by LPS mediated PMN activation (Fig. 1A) and PMN-platelet heteroaggregate formation (Fig. 1B^38^), which resulted in the increased amount of PMNs in thrombi. PMN attraction to the growing thrombi observed here has been previously demonstrated in vivo for another TLR4 agonist HMGB1^39^. Thus, LPS-induced PMN activation could be among the possible causes of bacteria-induced DIC.

Seeking the explanation of the disagreement with previously published works, we have performed an in vitro detailed analysis of the platelet functional responses. LPS were added to the whole blood. We avoided whole blood centrifugation in order to reduce the possible effects of ADP secreted by red blood cells in the process of platelet isolation. It appeared that LPS is capable of increasing calcium response upon 250 nM and 500 nM of ADP stimulation of platelets. LPS were capable of compensating ADP induced P2 receptor desensitization and restore the normal calcium and fibrinogen responses (Figs. S5, S6). It is noteworthy that the presence of sCD14 did not alter platelet responsiveness to LPS (Figs. S5–S7), thus assuming that sCD14 (as well as LBP) concentration in the blood plasma is sufficient for the TLR4 to transduce the signal inside of the cell^40^.

Seeking for the mechanism of such LPS action, we have performed additional analysis of platelet signaling upon pre-incubation of hirudinated PRP, collected avoiding buffy coat, with LPS. In these circumstances, it appeared that LPS could inhibit platelet responsiveness to low doses of ADP (500 nM and 1 µM, Figs. 3A–C and S8D–F, correspondingly) as well as a low dose of TRAP-6 (PAR1 receptor activating peptide, Fig. 3D–F), while not having any effect on CRP (collagen-related peptide) induced platelet responses (Fig. 3D–F). On the other hand, LPS was still capable of reducing the effects of 100 nM ADP induced desensitization (Fig. 3A–C). Hirudinated PRP aggregation was diminished by LPS as well (Figs. 4A,B, S9). It was shown that LPS is capable of inducing cGMP production and PKG activation in platelets^11^. However, it has been assumed that PKG activation by LPS appeared to enhance platelet activation instead of inhibiting it, which was in disagreement with other reports^21^. Thus, we analyzed the capability of LPS to induce cAMP/cGMP production and PKA/PKG activation. Inhibition of the PDEs by a low dose of IBMX did not impair platelet responsiveness to ADP (Fig. 3G,H). However, pre-treatment with LPS resulted in a synergistic enhancement of LPS mediated platelet inhibition (Figs. 3G,H, 4C). Interestingly, similar effects of PDE inhibition were observed in platelets incubated with physiological concentrations of nitrite^41^. Nitrite itself did not induce significant VASP phosphorylation; however, in combination with PDE inhibitor, VASP was strongly phosphorylated^41^. These data, together with our results, indicate that in platelets exist several complementary mechanisms in the regulation of PKA/PKG activity. On the other hand, direct measurement of PKA/PKG activity by VASP phosphorylation in response to LPS revealed that LPS impact was detectable, yet not statistically significant (Fig. 3I). Inhibition of the P2Y_1_ receptor by MRS2179 results in the enhanced sensitivity of platelet activation from cytosolic cAMP concentrations and, thus, the capability of LPS to abrogate MRS2179 treated platelets activation (Fig. S9E) also implies potential LPS-induced cAMP/cGMP rise in platelet cytosol.

Both PKA and PKG can inhibit PLCβ^42,43^ and IP3R^44,45^ activation and therefore act like inhibitors of platelet activation (Fig. 4C). However, it is noteworthy that these effects are detectable only upon weak stimulation. Nevertheless, in some conditions, LPS might be capable of activating PKG-mediated potentiation of platelet activation, which results in the highlighted discrepancy of the literature data. Such conditions should be investigated further.

Therefore, here we observed opposite effects of LPS on platelet activation: diminishing ADP induced desensitization (Figs. 3A–C, S5, S6) on the one hand and LPS induced inhibition of platelet activation on the other (Fig. 3A–F), which could be based on different mechanisms. The most obvious option is that LPS inhibits primary ADP response and thus, reduces desensitization. On the other hand, a weak increase in PKA activation upon pre-treatment by LPS also can lead to protein phosphatase 2A (PP2A) activation^46^, which is capable of dephosphorylating desensitized P2Y_1_ receptor, which leads to its rapid resensitization^30^.

Most of the reports on LPS-mediated platelet activation were based on the experiments with washed platelets^6,10^, whereas, in citrated PRP (calcium-free), LPS did not alter platelet aggregation^6^. However, based on our findings here, in the case of reversible platelet aggregation (Fig. S9), LPS is capable of inhibiting platelet activation. The nature of the reversible aggregation is contradictory. However, it has been demonstrated that it occurs only in the presence of calcium ions and upon weak stimulation^33^. Thus, it can be claimed that LPS induced PKA/PKG activation appears sufficient only for low doses of agonist.

In the process of platelet washing, red blood cells can secrete low doses of ADP, which results in platelet desensitization^47^. Furthermore, platelet aggregation is known to be highly dependent on secondary mediators of platelet activation (thromboxane A2 and ADP from the dense granules^10^) secretion of which might be diminished in desensitized platelets. Thus, the pre-incubation of platelets with LPS could compensate ADP induced platelet desensitization during platelet washing by the mechanism described above. This resulted in the increased platelet aggregation upon pre-incubation with LPS. The described mechanism enhances the role of the delicate handling of the cells during the washing process.

In summary, we observed the following effects of LPS on platelets: the LPS-mediated weak inhibition of platelet activation by a mechanism, potentially involving cAMP/cGMP signaling pathways (1) and the potentiation of platelet response to low doses of ADP (2). The latter could explain the enhancement of platelet activation upon LPS pre-treatment, described previously^5,10^.

## Materials and methods

### Materials

The sources of the materials were as follows: calcium-sensitive cell-permeable fluorescent dye Fura-Red-AM, (Molecular Probes, Eugene, OR); LPS O111:B4, LPS O127:B8, ADP, PGI_2_, EGTA, HEPES, bovine serum albumin, apyrase grade VII, TRAP-6 (SFLLRN) (Sigma-Aldrich, St Louis, MO); CD62p-Alexa647, CD11b-FITC, CD66b-PE (Sony Biotechnology, San Jose, USA); HBSS, RPMI 1640, Ficoll (PanEco, Moscow, RF), MRS2179, Mes-AMP (Tocris Bioscience, UK); Cysteine-containing version of cross-linked collagen-related peptide (CRP) was kindly provided by Prof. R.W. Farndale (the University of Cambridge, Cambridge, UK). Tyrode’s buffer (150 mM NaCl, 2.7 mM KCl, 1 mM MgCl_2_, 2 mM CaCl_2_, 0.4 mM NaH_2_PO_4_, 0.4 mM Na_2_CO_3_, 5 mM HEPES, 5 mM glucose, 0.5% BSA, pH 7.35) was fresh made from reagents (Sigma-Aldrich, St Louis, MO). IBMX was a kind gift of prof. S.P. Gambaryan (SPBU, St. Petersburg, Russia).

### Blood sample collection

Healthy volunteers, both sexes (23 males and 17 femails) aged between 18 and 35 years were recruited into the study. All of the donors claimed that they had neither inflammatory diseases, nor had taken any medication during at least two weeks prior to the assay. All of the donors were fasting at least 12 h before the experiment. Investigations were performed under the Declaration of Helsinki and written informed consent was obtained from all donors. The study was approved by the Independent Ethics Committee at CTP PCP RAS (1_2018-1 from 12.01.2018).

### LPS solution preparation

LPS were dissolved in deionised water or PBS to 10 mg/ml concentration. LPS aggregates were destructed by sonication for 30 times of 30 s cycles on ice. After sonication LPS were frozen in liquid nitrogen and stored att − 80 °C.

### PMN isolation and activation

PMNs were isolated from the whole blood collected in EDTA tubes. 6% Dextran was added in ratio 1:6. Then blood was incubated 30 min at 37 °C and 30 min RT after that. Leukocyte-rich plasma (LRP) was collected and mixed with hanks solution. Then the suspension was layered down on ficoll histopague 1.077. After centrifugation 400g 45 min + 4 °C the lower fraction was isolated and resuspended in hanks solution. Then PMNs were washed two times centrifugation 15 min 400g and resuspension in hanks solution. At the final stage neutrophils were resuspended in human serum (serum was obtained from blood, collected on coagulation activator and centrifugated sequentially at 2000g for 20 min and 2000g for 5 min).

PMNs were stimulated by LPS for 30 min at 37 °C; each sample was incubated with anti-CD11b and anti-CD66b antibodies. Cells were analysed by FACS Calibur flow cytometer (BD Biosciences, San Jose, USA).

### Heteroaggregate assay

PMN were washed as described above and resuspended in human serum. PRP from heparinised blood was obtained by centrifugation at 250g for 10 min, and only 1/3 from the upper part of the PRP was collected. PRP was added to the PMN suspension in human serum and incubated for 30 min in the presence of LPS or vehicle. Platelets were labeled by CD61-FITC, PMN by CD66b-PE antibodies and PMN heteroaggregate formation assays were performed by flow cytometry.

### Western blotting

Platelet washing and stimulation, SDS-PAGE and immunoblotting were performed using standard protocols as described previously^48^. Rabbit anti-human pIKκ-β (Cell Signal Technology, USA) was used to determine IKκ-β phosphorylation, rabbit anti-human PKC Substrate (Cell Signal Technology, USA) antibodies were used as a marker of platelet activation. Mouse anti-tubulin antibodies (Cell Signal Technology, USA) were used as a loading control. HRP-conjugated secondary antibodies were obtained from IMTEK, Russia. Blots were visualised using BioRad ChemiDoc XRS + Imaging System (Bio-Rad, Hercules, USA).

### Thrombus growth analysis

For thrombus growth assays blood was collected into SARSTEDT-Monovette hirudin (525 ATU/ml blood) tubes. Experiments were performed within 3 h after blood collection. Blood was supplied with 500 nM of DiOC6 and perfused in the PDMS flow chamber with collagen (Chrono-log, 0.2 µg/ml) covered glass with a syringe pump. The velocity of blood was adjusted to achieve the indicated wall shear rate, which was calculated from the dimensions of the chamber by the formula provided elsewhere^49^. For thrombus growth experiments low (200 s^−1^) and high (1,000 s^−1^) wall shear rates were utilised. Ten fields of view (82 × 82 μm each) were captured using Axio Observer Z1 microscope (Carl Zeiss, Jena, Germany) confocal microscope for each chamber at each analysed time point. Mean values of thrombus area were calculated for each donor than mean + -SEM values were calculated for n = 5 donors and presented on the figures. The height of the thrombi was calculated in the confocal regime by shifting the focal plane in the z-direction after 10 min of the experiment. For PMN activity, the experimental setting where PMNs are observed (low shear rate) were utilized^23^. Briefly, the wall shear rate was set at 100 s^−1^. Ten fields of view were observed by an inverted Nikon Eclipse Ti-E microscope (100 ×/1.49 NA TIRF oil objective) for three intervals of time (0–10 min, 10–20 min, 20–30 min), 30 fov in total. The activity of CD66b-positive cells was analysed by means of ImageJ software. The velocity of PMNs was calculated for each cell and then the mean values were calculated for each period of time for each donor (10–50 cells per period of time) and than the mean value and SEM for n = 5 donors was calculated.

### Aggregometry

Aggregation tests were performed in platelet-rich plasma and washed platelets. For platelet-rich plasma, blood was collected into tubes containing 3,8% sodium citrate. Blood was centrifugated at 150g 8 min. Only 2/3 of the PRP was collected, and the buffy coat was left intact in order to minimise potential contamination with PMNs which was less than 1% (data not shown). For preparation of washed platelets, blood was collected into 15 ml tubes containing 2.1 ml ACD. Platelets were purified by double centrifugation in the presence of PGI_2_ and hirudin and resuspended in Tyrode's albumin buffer with apyrase (0.01 U/ml) as described previously^47^.

Platelet aggregation was performed using Chrono-Log 490 aggregometer. LPS were added into samples under stirring conditions for 30, 60 or 120 min 37 °C. Aggregation of washed platelets was performed in the presence of 200 µg/ml of fibrinogen.

Hirudinated platelet plasma aggregation assays were performed using Biola LA-230. For platelet-rich plasma (PRP), blood was collected into tubes containing hirudin (SARSTEDT Monovette). Blood was centrifuged at 100g for 8 min without brakes. The experiments at Biola LA-230 were conducted in 200 μL aliquots of PRP with mixing by a stirrer at 800 rpm. PPP was used as a reference. ADP was added at various concentrations as the platelet activator. P2Y_1_ inhibitors MRS 2179 (100 µM) or P2Y_12_ inhibitor 2-MES-5-AMP (100 µM) were added in PRP 15 min before measurement and incubated for 15 min + 37 °C. Phosphodiesterase (PDE) inhibitor 3-Isobutyl-1-methylxanthine (IBMX, 10 µM) was incubated with PRP for 30 min + 37 °C before measurements. PRP was incubated with 100 μg/ml LPS or with PBS (vehicle) for 30 min + 37 °C without stirring before measurements.

### Low angle scattering (LaSca) analysis of the platelet diaggregate formation

Low angle scattering analysis of platelet activation was performed as described elsewhere^50^. Briefly, LASca is the method based on the measurement of low angle light scattering in a platelet suspension. This method was used to characterise the platelets function, allowing simultaneous recording of the formation of platelet diaggregates (light scattering by 1.5°) upon ADP stimulation. Platelet-rich plasma was diluted by Tyrode's buffer to 10,000 platelets/μl, stimulated by 50, 500 or 1,000 μM ADP, and the initial velocity and amplitude of aggregation at 1.5° and 12° were evaluated.

### Platelet flow cytometry assays

Platelet functional testing was performed similarly to^24^. Briefly, blood was collected in tubes with sodium citrate and diluted 1:20 in Tyrode's buffer. Diluted blood was either left intact or loaded with mepacrine. For platelet activation a mixture of CRP 20 µg/ml, PAR-1 activating peptide TRAP-6 (25 µM) and 5 mMCaCl_2_ was added to diluted blood in proportion 1:1 (10 + 10 µl) and incubated for 10 min. A mixture of antibodies was added to the activated and non-activated samples and incubated for 10 min. Samples were then diluted by adding 180 µl HBT buffer with CaCl_2_ and analysed by ACEA NovoCyte 3,000 cytometer.

For continuous flow cytometry assay blood was collected into SARSTEDT-Monovette hirudin (525 ATU/ml blood) tubes. Experiments were performed within 3 h after blood collection. Whole blood was dyed for 35 min at 37 °C by 2 µM of Fura-RED in the presence of 0.5 U/ml of apyrase. Then the blood was diluted 50 times in Tyrode's buffer and rested 30 min at 37 °C. For flow cytometry, the samples were further diluted to achieve low platelet concentration (1 × 10^6^ cells/ml final) and analysed using BD FACS Canto II flow cytometer. Platelet cytosolic calcium concentration was calculated from Fura-red fluorescence upon excitation with 405 nm and 488 nm based on the Grynkiewicz formula^51^ upon calibration by 1 µM of ionomycin and 10 mM of EGTA.

### Data processing and statistical analysis

Fluorescence microscopy data was assessed using ImageJ software. PMN crawling velocity was calculated using the Fiji manual tracking plugin. Flow cytometry data were analysed by means of FlowJo (https://www.flowjo.com/) and python 3.7. Statistical analysis of the data was performed by means of Python 3.6. Western blots were analysed using ImageJ software.

## Supplementary information

Supplementary Figures.
