# Supplementary material for: Effects of bacterial lipopolysaccharides on platelet function: inhibition of weak platelet activation
Source: Sci Rep. 2020 Jul 23;10:12296. doi: 10.1038/s41598-020-69173-x (PMC7378070; doi:10.1038/s41598-020-69173-x)
Supplement: Supplementary file 1 — Supplementary Figures. [file 41598_2020_69173_MOESM1_ESM.pdf]

Supporting Information

Effects of bacterial Lipopolysaccharides on platelet function: inhibition of weak platelet activation

Alexey A. Martyanov[1,2,3,4]*, Aleksandr S. Maiorov[1], Aleksandra A. Filkova[1,2,4], Elena O. Artemenko[1,2], Galina A. Svidelskaya[4], Alexander A. Ryabykh[1,2], Stepan P. Gambaryan[5], Mikhail A. Panteleev[1,2,4] and Anastasia N. Sveshnikova[1,2,4,6]

1 Center for Theoretical Problems of Physico-Chemical Pharmacology, Russian Academy of Sciences, 30 Srednyaya Kalitnikovskaya str., Moscow, 109029, Russia

2 National Medical Research Center of Pediatric Hematology, Oncology and Immunology named after Dmitry Rogachev, 1 Samory Mashela St, Moscow, 117198, Russia

3 Institute for Biochemical Physics (IBCP), Russian Academy of Sciences (RAS), Russian Federation, Moscow, Kosyigina 4; 119334, Russia

4 Faculty of Physics, Lomonosov Moscow State University, 1/2 Leninskie gory, Moscow, 119991, Russia

5 Sechenov Institute of Evolutionary Physiology and Biochemistry, Russian Academy of Sciences, St. Petersburg 194223, Russia

6 Department of Normal Physiology, Sechenov First Moscow State Medical University, 8/2 Trubetskaya St., Moscow, 119991, Russia

*To whom correspondence should be addressed: Alexey A. Martyanov, aa.martyanov@physics.msu.ru, https://orcid.org/0000-0003-0211-6325.

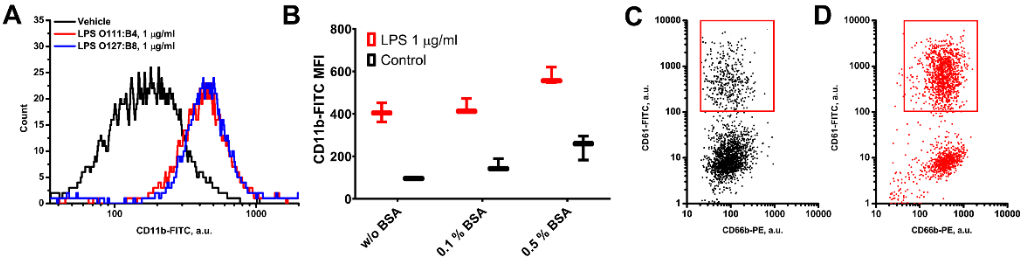

Figure S1. PMN activation by LPSs. (A) Preincubation with LPS resulted in the increase in CD11b activation on PMNs. (B) Introduction of BSA did not significantly affect LPS mediated increase in PMN CD11b activation. (C,D) Typical dot-plots of resting platelet-PMN suspension (C) and pre-incubated with LPS (D).

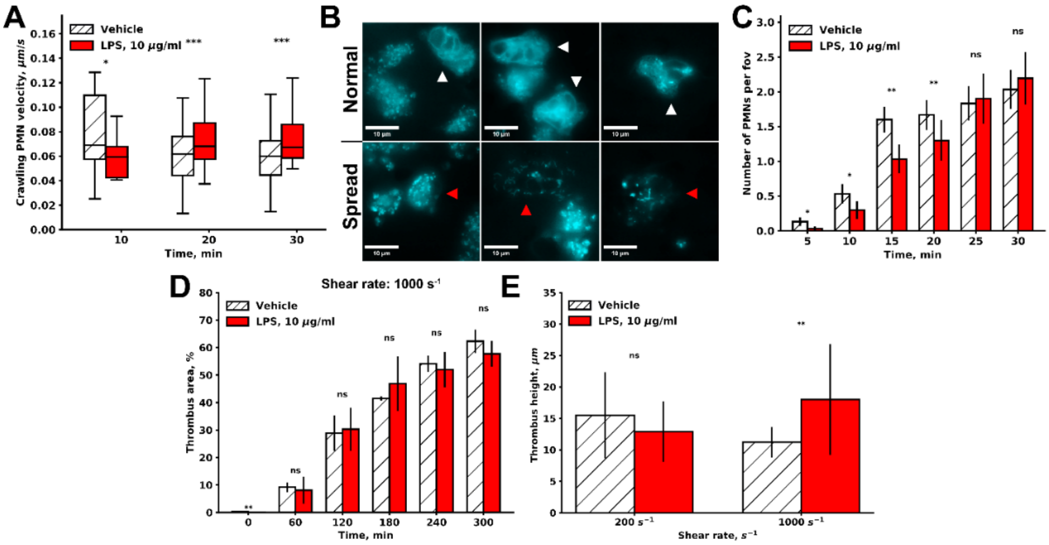

Figure S2. Parallel-plate flow chamber whole blood experiments. (A) LPSs slightly increased the velocity of crawling PMNs on fibrillar collagen and significantly increased the amount of highly spread PMNs (B); (C) LPS' effects on the number of PMNs per field of view were limited to a slight decrease at the early stages of thrombus formation; (D) Pre-incubation with LPS did not affected thrombus area at wall shear rate 1000 s^{-1} ; (E) LPSs slightly increased the average height of the growing thrombi at 1000 s^{-1}, while there was no significant impact at 200 s^{-1}. Bars represent mean, whiskers represent SEM. Significance was calculated by Mann-Witney test, n = 5, * - p < 0.05, ** - p < 0.01, *** - p < 0.001.

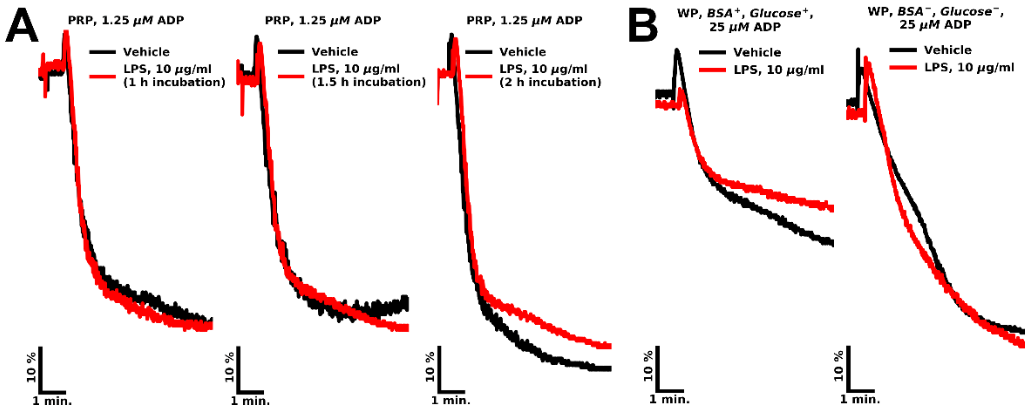

Figure S3. Preincubation of platelets with LPS did not alter platelet aggregation. Light transmission aggregometry, typical curves out of n = 10. (A) Platelet rich plasma from citrated blood. (B) Washed platelets in BSA^+, $Glucose^+$ and BSA^-, $Glucose^-$ Tyrode's buffer.

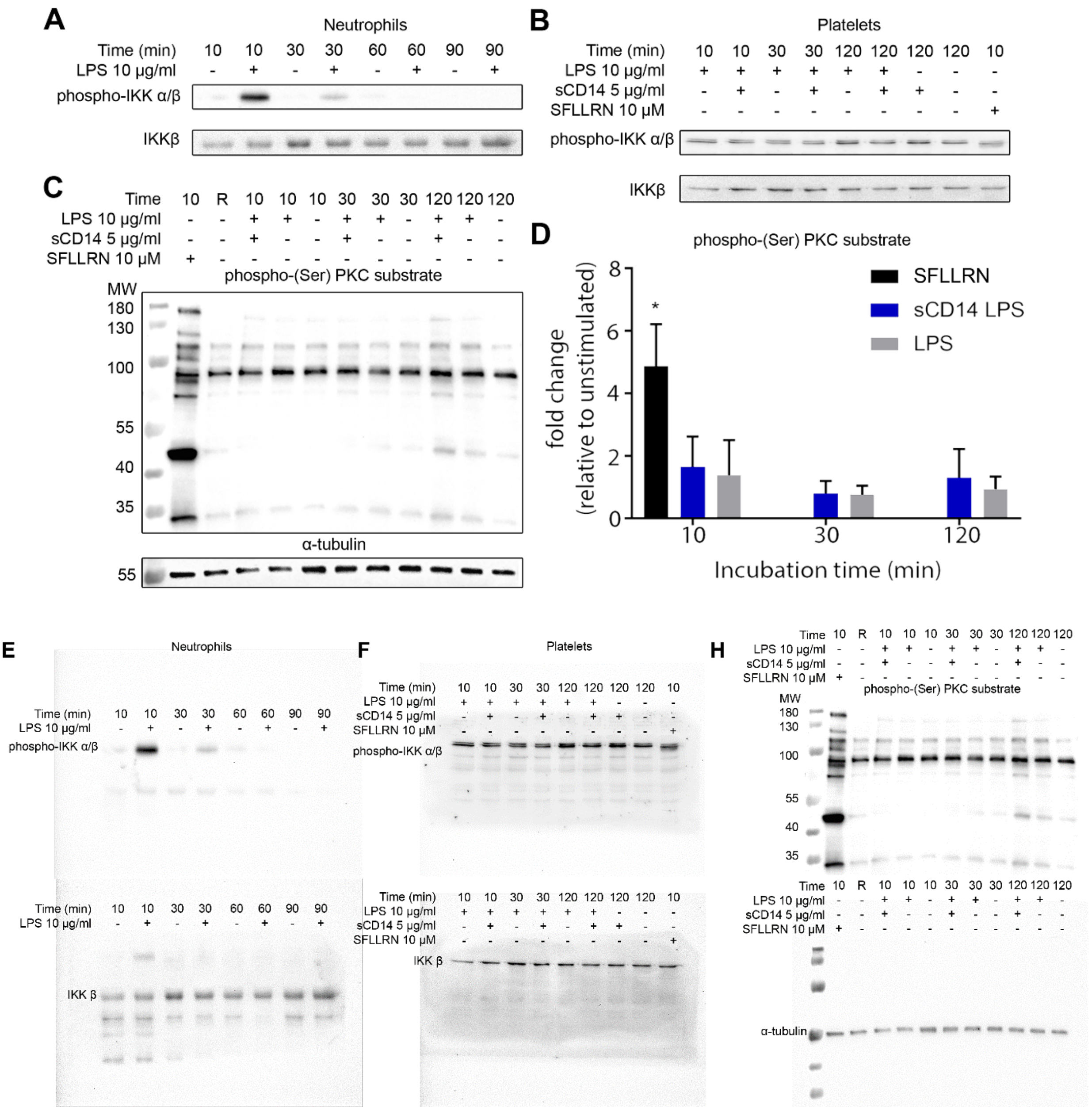

Figure S4. IKκ phosphorylation in response to LPSs. (A) 10 minute pre-incubation with 10 µg/ml LPS increased IKκ α/β phosphorylation, which decreased with the flow of time. Loading control was assessed using anti IKκ α/β antibodies after membrane stripping. (B) Neither 10 µM of SFLLRN (TRAP-6) nor 10 µg/ml LPS in the presence and absence of sSCD14 affected IK IKκ α/β phosphorylation in platelets. Loading control was assessed using anti IKκ α/β antibodies after membrane stripping. (C,D) Activation of platelets by 10 µg/ml LPS or 10 µg/ml LPS and sCD14 was non-significant in comparison to 10 µM of SFLLRN (TRAP-6). Full length blots are provided in figure S6. (E-H). Full length western blots. (E) IKκ α/β phosphorylation in neutrophils after incubation with LPS for different time intervals. IKκ β was used as loading control. (G) IKκ α/β phosphorylation in neutrophils after incubation with LPS for different time intervals or activation by 10 µM of SFLLRN (TRAP-6). IKκ β was used as loading control. (H) Ser phosphorylation (PKC substrate) in platelets activated by 10 µM of SFLLRN (TRAP-6) or 10 µg/ml of LPS for different time intervals. α-tubulin was used as loading control.

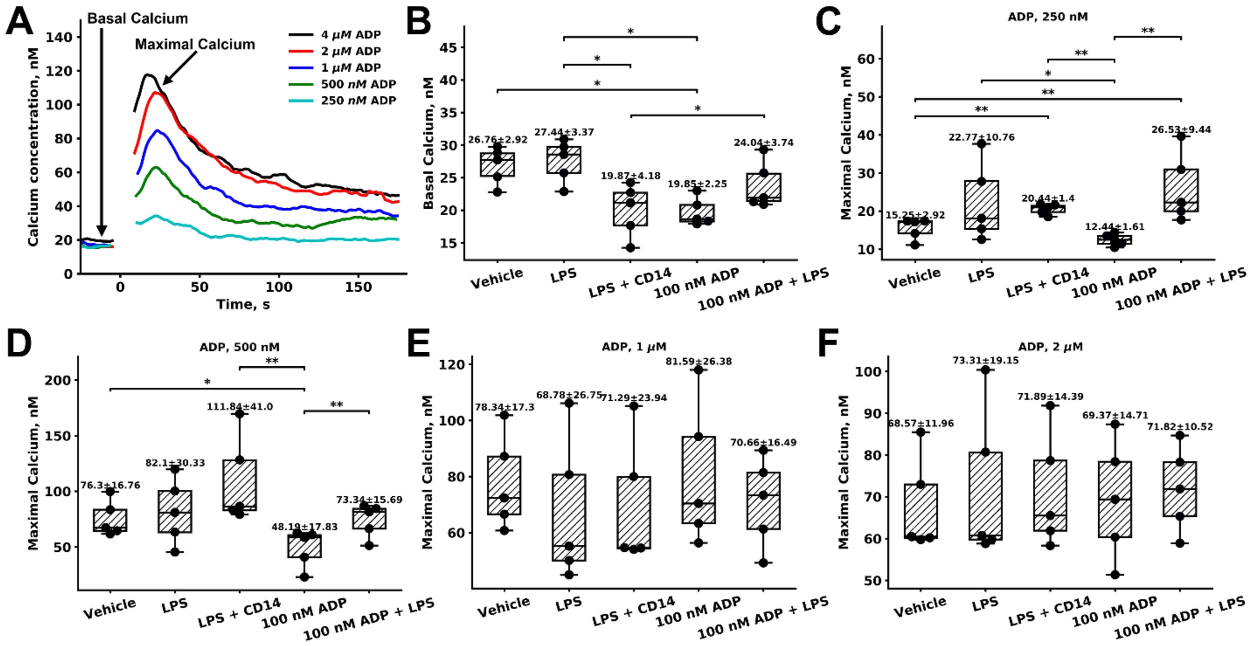

Figure S5. LPS effects on platelet calcium signalling. (A) Typical platelet suspension calcium response to ADP at different concentrations. (B) Basal calcium concentration in resting platelets upon pre-incubation with vehicle, 10 µg/ml LPS, 10 µg/ml LPS and 5 µg/ml sCD14, 100 nM ADP, 100 nM ADP and 10 µg/ml LPS for 30 minutes. (C-F) Calcium mobilization (Maximal calcium concentration minus basal calcium concentration) for platelets stimulated by 250 nM (C), 500 nM (D), 1 µM (E) and 2 µM (F) of ADP. Significance was calculated by Mann-Witney test (* - p < 0.05; ** - p < 0.01; *** - p < 0.001; n = 5).

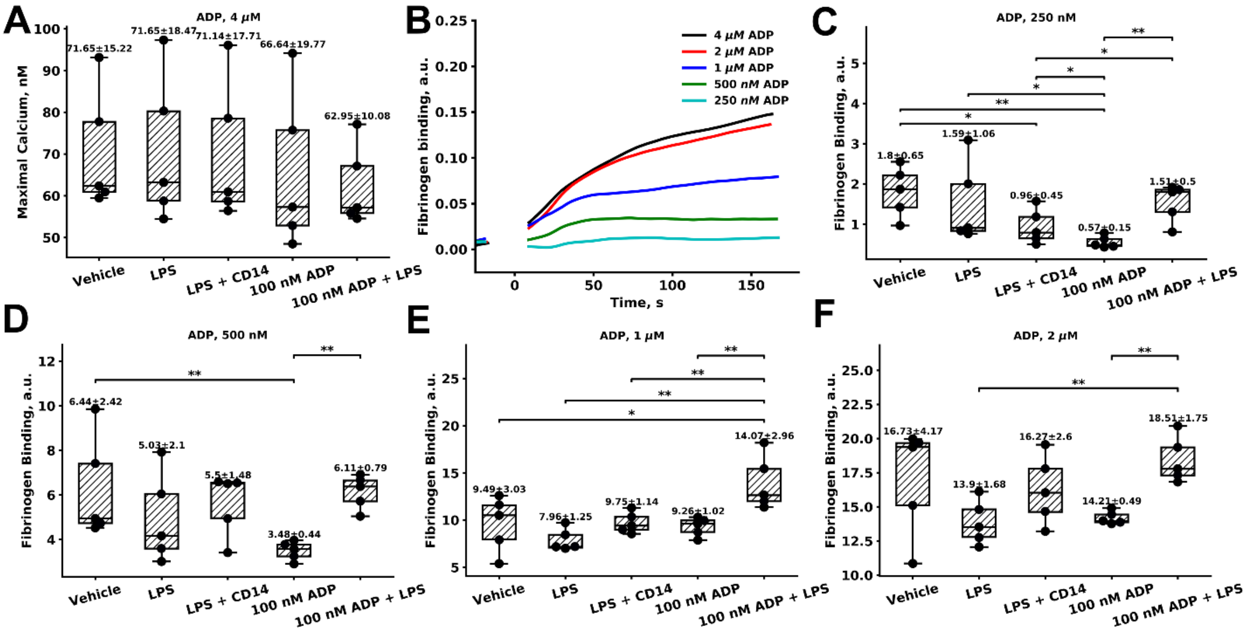

Figure S6. (A) Maximal calcium concentration upon stimulation by 4 µM of ADP. (B) Characteristic fibrinogen binding changes upon stimulation of platelets by different concentrations of ADP in continuous flow cytometry experiments. (C-F) Fibrinogen binding upon pre-incubation of platelets with vehicle, 10 µg/ml LPS, 10 µg/ml LPS and 5 µg/ml sCD14, 100 nM ADP, 100 nM ADP and 10 µg/ml LPS activated by 250 nM (C), 500 nM (D), 1 µM (E) and 2 µM (F) of ADP. Significance was calculated by Mann-Witney test (* - p < 0.05; ** - p < 0.01; *** - p < 0.001; n = 5).

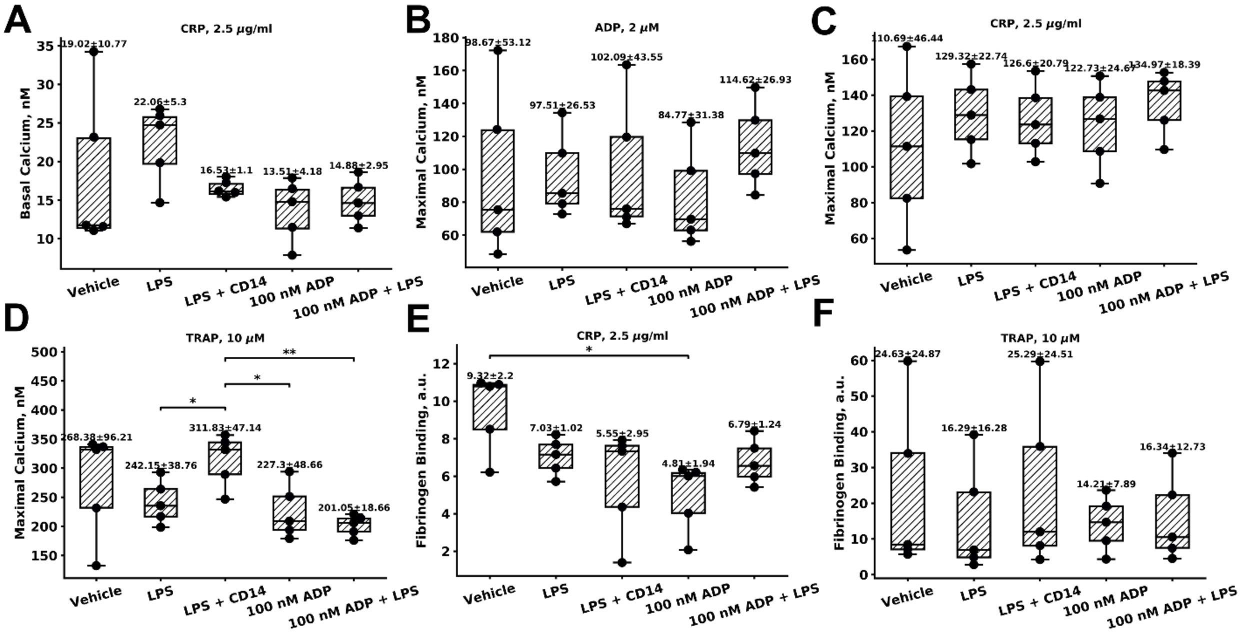

Figure S7. (A) Basal calcium concentration in resting platelets upon pre-incubation with vehicle, 10 µg/ml LPS, 10 µg/ml LPS and 5 µg/ml sCD14, 100 nM ADP, 100 nM ADP and 10 µg/ml LPS for 2 h. (B-D) Changes in calcium concentration (Maximal Calcium – Basal Calcium) for platelets stimulated by 2 µM of ADP (B), 2.5 µg/ml of CRP (C) and 10 µM of SFLLRN (D) at different conditions in the continuous regime. (E,F) Maximal fibrinogen binding for platelets stimulated by 2.5 µg/ml of CRP (E) and 10 µM of SFLLRN (F) at different conditions in the continuous regime. Significance was calculated by Mann-Witney test (* - p < 0.05; ** - p < 0.01; *** - p < 0.001, n = 5).

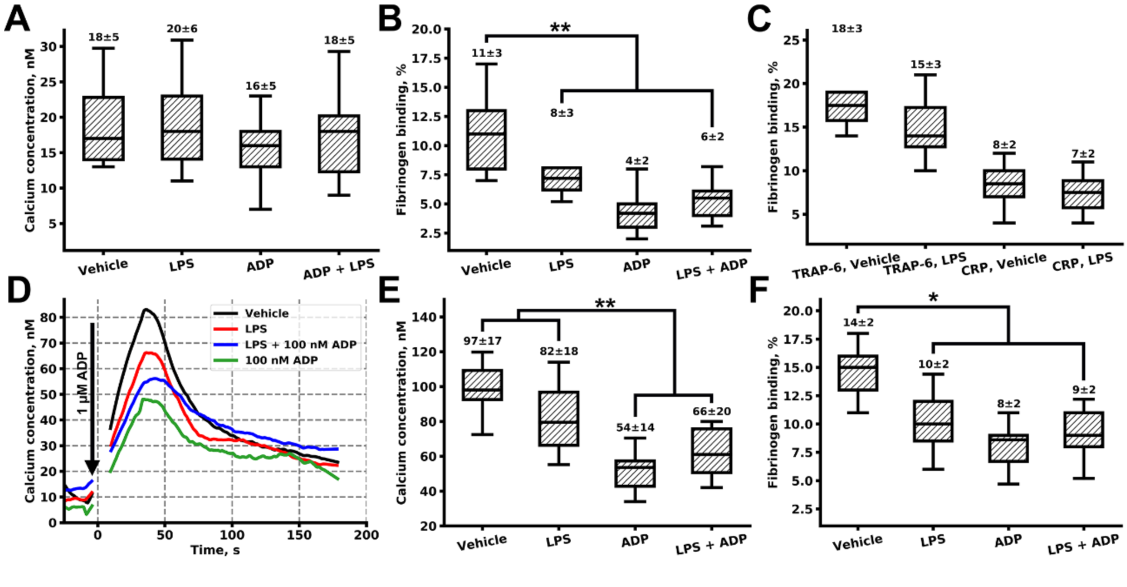

Figure S8. (A) Basal calcium concentration in resting platelets upon pre-incubation with vehicle, 100 µg/ml LPS, 100 nM ADP or 100 nM ADP and 100 µg/ml LPS. (B) Fibrinogen binding by platelets upon pre-incubation with vehicle, 100 µg/ml LPS, 100 nM ADP or 100 nM ADP and 100 µg/ml LPS and activation by 500 nM ADP. (C) Fibrinogen binding by platelets upon pre-incubation with vehicle or 100 µg/ml LPS and activation by 1 µM TRAP-6 or 1 µg/ml CRP. (D) Typical cytosolic calcium concentration curves upon activation by 1 µM ADP after pre-incubation with 100 µg/ml LPS, 100 µg/ml LPS and 100 nM ADP, 100 nM ADP or vehicle. (E,F) Pretreatment with LPS and ADP or sole ADP resulted in a significant decrease in a calcium (E) and fibrinogen (F) responses to 1 µM ADP (n = 15 donors, significance was calculated by Mann-Whitney test, * - p < 0.05; ** - p < 0.01; *** - p < 0.001).

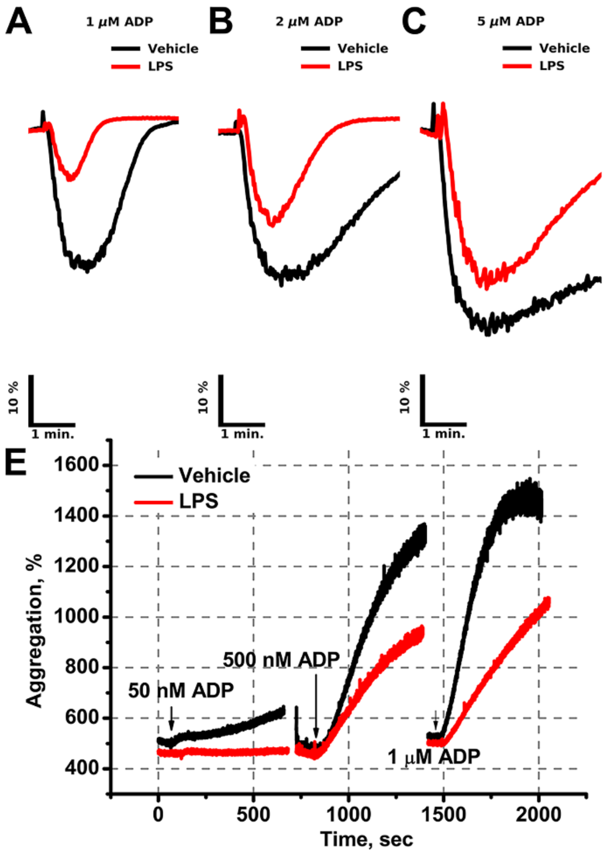

Figure S9. LPS mediated alterations in platelet aggregation. Pre-treatment with 100 µg/ml of LPS resulted in a decrease in platelet aggregation in response to 1 µM (A), 2 µM (B) and 5 µM of ADP (C). Platelet di-aggregate formation was impaired by LPS upon stimulation by all concentrations of ADP tested (E).
